# Spatial Proximity of LAG‐3 and T Cell Receptor: A New Paradigm for T Cell Suppression

**DOI:** 10.1002/mco2.70533

**Published:** 2025-11-28

**Authors:** Xiaoqi Miao, Xie Feng, Fangfang Zhou

**Affiliations:** ^1^ Department of Ultrasound, the First Affiliated Hospital, the Institutes of Biology and Medical Sciences, Suzhou Medical College Soochow University Suzhou Jiangsu China; ^2^ The First Affiliated Hospital, the Institutes of Biology and Medical Sciences, Suzhou Medical College Soochow University Suzhou Jiangsu China

1

In a recent study published in Cell, Du et al. reveal that the inhibitory function of the immune checkpoint LAG‐3 is strictly guided by its spatial proximity to the T cell receptor (TCR), independent of CD4. This groundbreaking research clarifies a long‐standing theoretical gap in T cell suppression and pioneers a novel bispecific T cell silencing antibody (BiTS) therapy, offering a transformative paradigm for treating T cell‐driven autoimmune diseases [1].

As a homolog of CD4, LAG‐3 has become a research focus due to its association with CD4 in MHC‐II interactions and T cell signaling. Historically, it was believed that LAG‐3 blocks T cell activation signals by competitively inhibiting the binding of CD4 to MHC‐II and weakens TCR signaling by disrupting the interaction between CD4 and Lck. Previous studies suggested that its inhibitory function is highly dependent on CD4 [2, 3]. Meanwhile, LAG‐3 has a unique action pathway in CD8⁺T cells, and studies have confirmed that targeting LAG‐3 can restore the effector function of CD8⁺T cells through the CD94/NKG2‐Qa‐1b signaling pathway [4]. A key breakthrough of this study is the clear decoupling of LAG‐3's inhibitory function from CD4 co‐receptor activity. Through precise experiments including CRISPR‐mediated CD4 knockout and reconstitution with chimeric CD4‐Lck proteins (where Lck is constitutively anchored), researchers found that LAG‐3's inhibitory effect persists. This result elegantly demonstrates that LAG‐3 functions independently of CD4 signaling or disruption of CD4/Lck interaction, challenging the prior belief in CD4 dependence and opening new research avenues.

To further explore LAG‐3's inhibitory mechanism, researchers employed engineered artificial antigen‐presenting cells (aAPCs) expressing both membrane‐anchored anti‐CD3 single‐chain antibodies (scFv) to activate TCR signals and non‐cognate pMHC‐II (binding LAG‐3 but not TCR). Under these conditions, LAG‐3's T cell inhibition completely vanished. This decoupling of MHC‐II/LAG‐3 binding from TCR activation entirely blocked LAG‐3 function, in stark contrast to the still‐active PD‐1/PD‐L1 pathway in the same system. This striking comparison strongly proves that LAG‐3 requires cognate pMHC‐II‐bridged spatial proximity to TCR. The researchers further verified spatial proximity's critical role in LAG‐3 function. This functional reconstruction crucially confirms that TCR proximity is an indispensable triggering factor for LAG‐3, experimentally supporting spatial proximity as its key inhibitory mechanism. This is consistent with the mechanism revealed in relevant studies, which found that when LAG3 binds to its main ligand MHC‐II, it triggers monoubiquitination of specific lysine residues in the intracellular segment, and this ubiquitination can hinder the membrane sequestration of the EP motif, exposing it and initiating inhibitory signal transduction [5].

This study is the first to clarify that LAG‐3 directly interferes with TCR signaling through its intracellular domain (ICD): its inhibitory function strictly depends on pMHC‐II‐mediated spatial colocalization of TCR and LAG‐3, independent of CD4. Specifically, LAG‐3's ICD uses the FSAL motif (F483/L486) to specifically bind CD3ε’s basic‐rich sequence (BRS), competitively blocking interaction between activated Lck and doubly phosphorylated CD3ε—an effect not seen with PD‐1/CTLA‐4. Motif mutation validation and TCR/Lck colocalization experiments further confirm that LAG‐3 inhibits CD3ε‐Lck signaling via this molecular competition, offering new targets for immunotherapy.

Based on these findings, researchers developed LAG‐3/TCR‐targeting bispecific T cell silencing antibodies (BiTS) (Figure 1). These antibodies fuse anti‐mouse TCRβ (H57‐597) and anti‐LAG‐3 (M8) single‐chain variable fragments (scFv) with an N297G mutation to eliminate Fc effector functions. Three controls ensured specificity: CDR3 heavy chain‐mutated BiTSmut (losing LAG‐3 binding), lysozyme‐substituted control (Ly/TCR BiTS), and humanized BiTS (based on relatlimab).In T cell hybridomas, BiTS achieved potent LAG‐3‐dependent IL‐2 inhibition in CD4⁺3A9 cells stimulated with cognate pMHC‐II, CD8⁺B3Z cells with pMHC‐I, and CD4/CD8 knockout models, without interfering with MHC‐I tetramer binding. These results confirm BiTS bypass CD4⁺ restrictions via forced LAG‐3‐TCR cis‐proximity, enabling co‐receptor‐independent broad T cell inhibition. Experiments on primary CD8⁺ T cells (from OT‐I×Lag3−/− mice) showed BiTS dose‐dependently inhibited OVA_257_₋_264_ peptide‐induced IL‐2 and IFN‐γ secretion, with this effect abrogated in Lag3−/− cells or with low‐dose BiTSmut. Bulk RNA sequencing further confirmed BiTS downregulates T cell activation genes such as *Ifng* and *Il23a*, validating inhibition at the molecular level.

**FIGURE 1:**
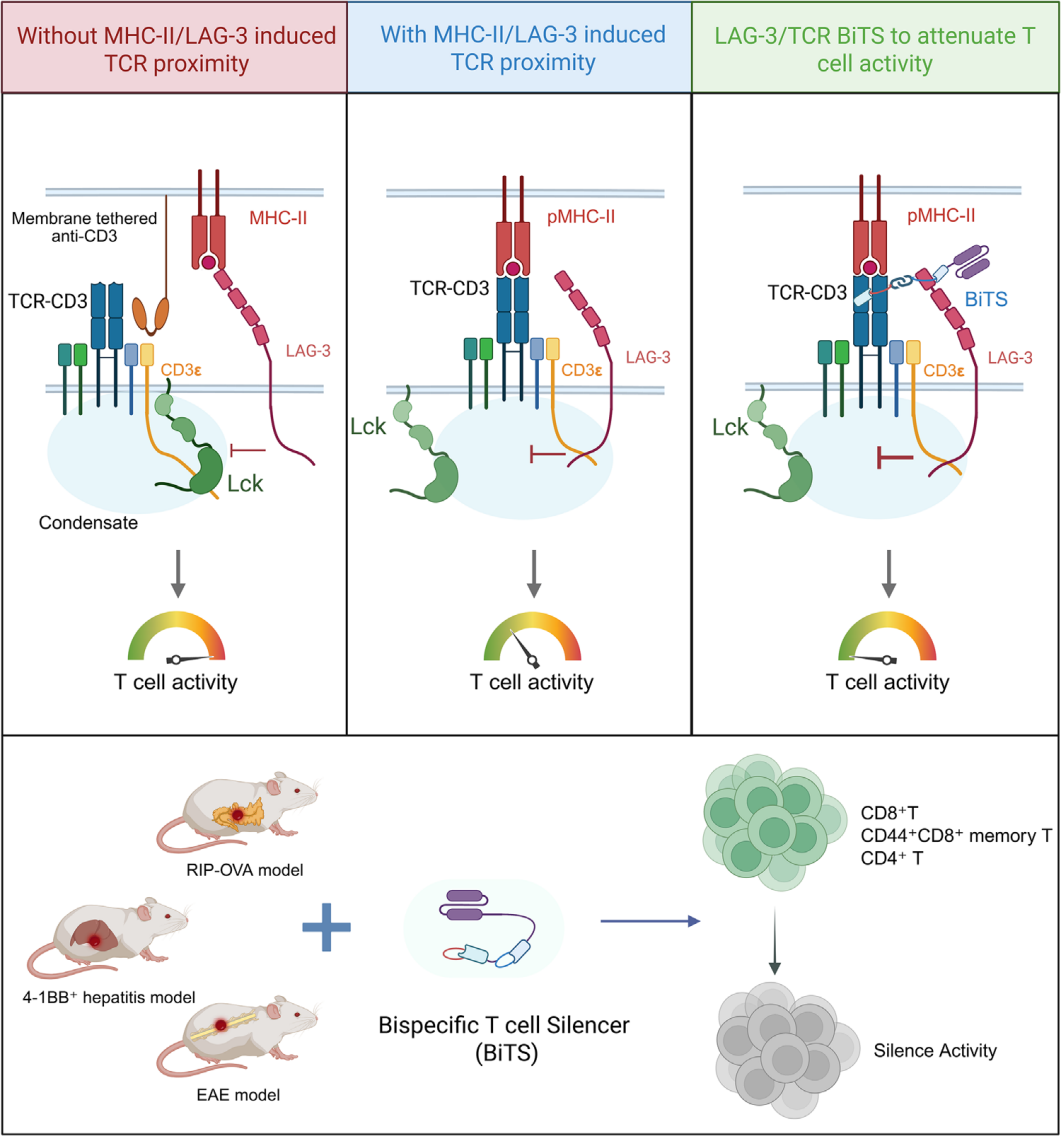
Diagram of TCR‐LAG‐3 spatial proximity regulation and BiTS intervention mechanism. Left area: No MHC‐II/LAG‐3‐induced TCR proximity—Membrane‐anchored anti‐CD3 antibodies activate TCR signals, but in the absence of pMHC‐II bridging, LAG‐3 is spatially segregated from TCR, failing to form inhibitory phase‐separated complexes, and T cell activity remains normal. Central area: MHC‐II/LAG‐3‐induced TCR proximity—Homologous pMHC‐II bridges TCR and LAG‐3, and forced spatial proximity drives LAG‐3 ICD and CD3ε to form dynamic condensates, competitively blocking Lck activation and inhibiting T cell activity. Right area: BiTS therapy simulates spatial proximity to inhibit T cells—Bispecific T cell silencing antibodies (BiTS) forcibly bring the TCR‐LAG‐3 distance closer through anti‐TCRβ/anti‐LAG‐3 scFv, achieving constitutive inhibition independent of pMHC‐II. Inset (bottom right): Efficacy verification in disease models—BiTS significantly inhibit pathogenic T cell activity, reduce islet CD8⁺ T cell infiltration in diabetes models, clear intrahepatic CD8⁺ T memory (Tm)/tissue‐resident memory T (TRM) subsets in hepatitis models, and inhibit CD4⁺ T cell‐mediated neuroinflammation in the central nervous system of EAE models. Created in BioRender. xiaoqi, m. (2025) https://BioRender.com/imem9tx BiTS (Bispecific T cell silencing antibodies); EAE (Experimental Autoimmune Encephalomyelitis); LAG‐3 (Lymphocyte Activation Gene‐3); Lck (Lymphocyte‐specific protein tyrosine kinase); MHC‐II (Major Histocompatibility Complex Class II); pMHC‐II (Peptide‐MHC Class II); RIP‐OVA model (NOD8.3/RIP‐OVA T1D model); TCR (T Cell Receptor); TRM (Tissue‐Resident Memory T cell).

In disease models, BiTS showed significant therapeutic potential. In the RIP‐OVA autoimmune diabetes model, 0.75 mg/kg injections every 2 days reduced diabetes incidence, pancreatic CD8⁺ T cell infiltration, and islet inflammation (absent with BiTSmut), confirming LAG‐3‐dependent inhibition of antigen‐specific CD8⁺ T cell responses to prevent type 1 diabetes progression. In a mouse model mimicking “activated CD8⁺ T cell hepatitis” in idiopathic neonatal acute liver failure (IND‐PALF), BiTS reduced hepatic CD8⁺ T cell infiltration, lowered inflammatory cytokines (e.g., IFN‐γ, TNF‐α), alleviated liver damage, and inhibited activation/function of pathogenic CD8⁺ T cells (especially central memory and tissue‐resident memory subsets). In the CD4⁺ T cell‐mediated multiple sclerosis model (EAE), BiTS also reduced clinical scores. These results confirm BiTS's broad therapeutic potential for various CD8⁺ and/or CD4⁺ T cell‐driven autoimmune diseases.

This study not only conducts an in‐depth analysis of the precise regulatory mechanism of LAG‐3 as a conditional immune checkpoint, challenging the traditional cognition that it is dependent on CD4, and clarifies the core role of spatial proximity in its inhibitory function, but also pioneeringly proposes a transformative therapeutic paradigm targeting the spatial proximity of LAG‐3/TCR (cis‐targeting). The developed BiTS technology offers immense clinical application prospects for T cell‐driven autoimmune diseases. By forcibly inducing LAG‐3/TCR proximity in cis, BiTS can selectively silence pathogenic T cells independent of MHC‐II and CD4. This mechanism holds promise for overcoming the limitations of conventional broad immunosuppressants, offering a targeted approach with reduced systemic toxicity. Furthermore, the successful in vivo suppression across multiple mouse models, including T1D (RIP‐OVA), hepatitis, and EAE, suggests a broad applicability in human conditions where pathogenic T cells are dominant. The potential for a targeted T cell “silencer” is expected to bring good news to the majority of patients in future clinical practice. At the same time, the research also provides new ideas and methods for in‐depth exploration in the field of immune checkpoints, promoting the field to develop in a more precise and in‐depth direction.

## Author Contributions

Xiaoqi Miao wrote the manuscript and prepared the figure. Feng Xie provided valuable discussion. Feng Xie and Fangfang Zhou approved the final version of the manuscript. All authors have read and approved the final manuscript.

## Funding

This work was supported by the Chinese National Natural Science Funds (92574101, 82473119) and a project Funded by the Priority Academic Program Development of Jiangsu Higher Education Institutions.

## Conflicts of Interest

The authors declare no conflicts of interest.

## Ethics Statement

The authors have nothing to report.

## Data Availability

The authors have nothing to report.
